# Cutaneous Lesions as the Sole Presentation of Tuberous Sclerosis

**DOI:** 10.7759/cureus.9528

**Published:** 2020-08-02

**Authors:** Mohanad Ahmed, Khalid Hassan, Mohamed Elfatih Mustafa

**Affiliations:** 1 Internal Medicine, Hamad Medical Corporation, Doha, QAT; 2 Medicine, Hamad Medical Corporation, Doha, QAT

**Keywords:** tuberous sclerosis, hamartoma, angiomyolipoma’s

## Abstract

Tuberous sclerosis complex (TSC) is a rare genetic, neurocutaneous condition characterized by hamartomas in different organs, including the brain, skin, heart, kidney, and lungs. Fibromas are the typical presentation, but rare symptoms may present as well. We present the case of a 26-year-old woman who presented to our clinic with long-standing cutaneous manifestations of TSC and lacked the typical neurological and intellectual signs of the condition.

## Introduction

Tuberous sclerosis complex (TSC) is a genetic disease with a prevalence of nine in 100,000 cases [1] discovered in the mid-1700s via postmortem investigations [2]. This rare neurocutaneous syndrome presents with glial tumors in the brain and retina as well as fibromas in the skin and other organs [3]. TSC is characterized by a triad of epilepsy, intellectual disability, and adenoma sebaceous [4]. Approximately 50% of patients present with normal intellect, and 15% present with no seizures [5]. Neurological presentations are often seen in children, while cutaneous manifestations occur later in adult life. This report describes the case of a 26-year-old woman who presented to our clinic with cutaneous manifestations of TSC but did not have the typical neurological and intellectual signs of the condition.

## Case presentation

A 26-year-old woman presented to the general medicine clinic with a growing painless mass on her right and left index fingers and right middle toe. She also had a fibroma on her chin and hypopigmentation on her upper back. She reported having these signs for four years. The patient is a university graduate where she studied management with good scores. She did not experience any neurological symptoms, including seizures. She had no family history of similar conditions and no family history of seizures. She has normal menstruation and no history of surgeries. Her medical history was significant for chronic iron-deficiency anemia.

**Figure 1:**
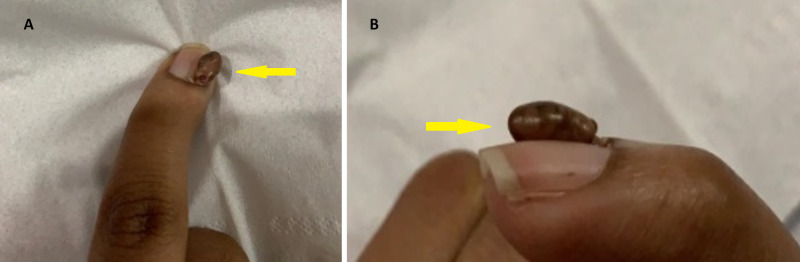
Sub-angular fibroma in (A) posteroanterior view and (B) lateral view

**Figure 2:**
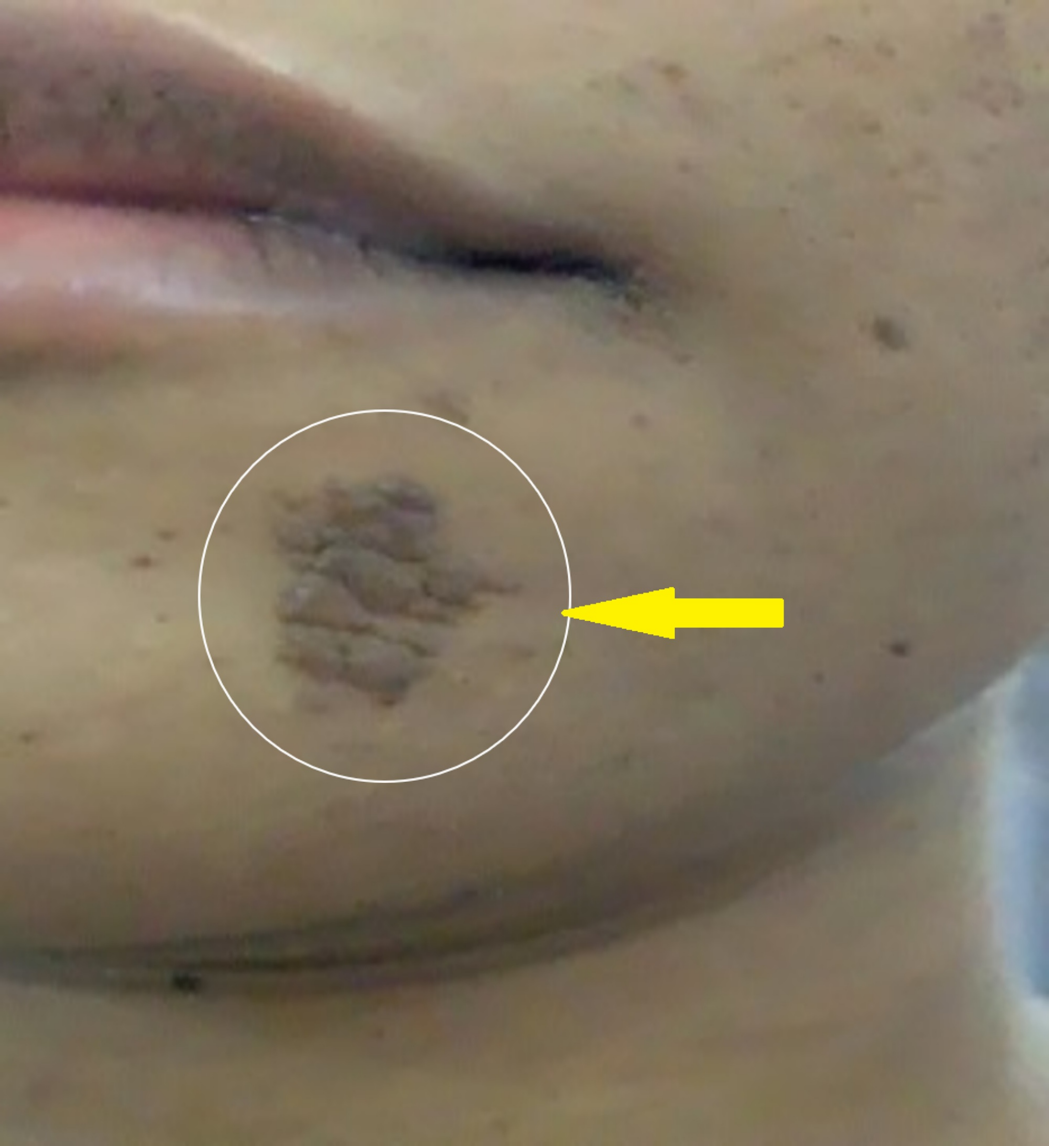
Chin fibroma (white circle)

On examination, we noted stable vital signs and normal intelligence quotient. We noted a subangular fibroma (Figure 1) as well as fibromas on her chin and back (Figure 2). The results of all other examinations were unremarkable, including eyes and neurological assessments.

**Figure 3:**
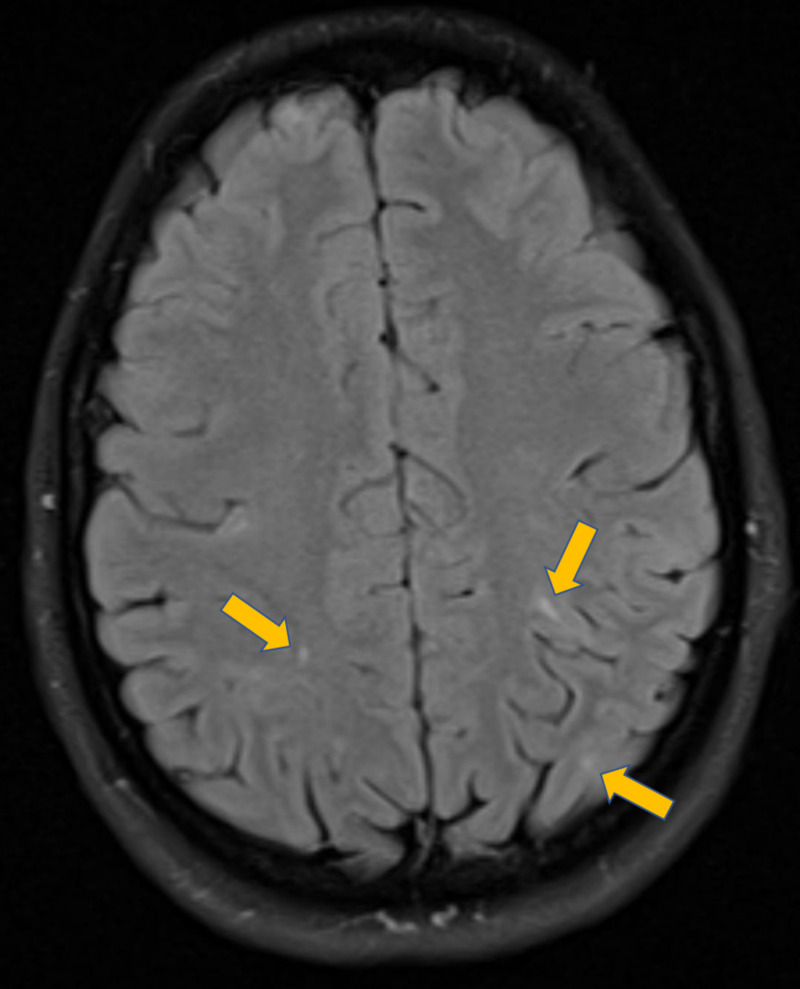
MRI brain showing multiple tuberii MRI: magnetic resonance imaging.

**Figure 4:**
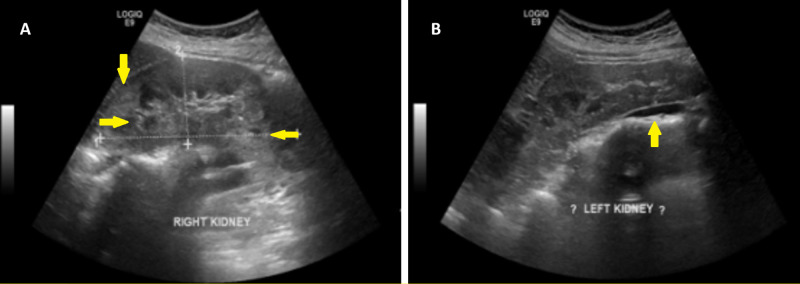
Ultrasound revealing multiple bilateral renal angiomyolipomas in the (A) right kidney and (B) left kidney

Her hematological profile showed evidence of iron deficiency anemia. A magnetic resonance image of her brain showed multiple tuberii (Figure 3), and an abdominal ultrasound revealed multiple bilateral renal angiomyolipomas (Figure 4).

Genetic screening is important to complete the picture, but due to the cost of the test, and given that the patient is visiting from another country, genetic screening was not performed. The patient was referred to the neurology team for further consultation and follow-up.

## Discussion

TSC is a rare autosomal dominant genetic disorder caused by mutations in two separate genes, TSC1 and TSC2 [6]. The average age at diagnosis is seven years. Most cases (81%) are diagnosed before the age of 10, but diagnosis during adolescence and adulthood is not uncommon [7]. The most common presenting symptoms and signs include new onset of seizures, history of seizures, infantile spasms, family history of TSC, cardiac rhabdomyomas, and hypopigmented macules [7]. Most patients with TSC have epilepsy, and approximately 50% have cognitive deficits and learning disabilities [6].

Comparing our case against the general knowledge in the literature, our patient's presenting symptoms are not typical for TSC diagnosis, given that new-onset seizure and family history of seizure are not present in our case. However, an absence of a family history of the disease is atypical in TSC because TSC is inherited in an autosomal dominant pattern. Our patient's age at presentation is also atypical.

The diagnosis of TSC is based on genetic testing and clinical criteria [6]. Identifying either a TSC1 or TSC2 pathogenic mutation is sufficient for the diagnosis of TSC. Genetic testing is not required for patients who fulfill clinical criteria for definite TSC, but it is useful for confirming the diagnosis in individuals with possible TSC for reproductive planning and identifying at-risk family members [6].

Our patient presented only with a growing painless mass in her fingers, and in most of her visits to primary health care centers, her health care providers assured her the lesions were benign and required no further management other than regular follow-up assessments. The patient spent more than two years under follow-up observation absent any treatment, with no suspicion or workup for TSC given the absence of other classical presenting features and family history. Because of her two-year history and our suspicion of TSC, we ordered tests for TSC, which showed multiple tuberii in the central nervous system and renal angiomyolipoma. The patient was diagnosed clinically based on the presence of two significant clinical findings after the workup.

Upon reviewing the literature, we found many reported cases of TSC that are misdiagnosed. One young girl was misdiagnosed as neurofibromatosis because of her cutaneous lesions [3], and another patient's TSC was misdiagnosed as epilepsy [8].

## Conclusions

We presented the case of a young girl with cutaneous manifestations and radiological indications of TSC, which was initially misdiagnosed as benign skin lesions. This case serves as a reminder for physicians to retain a high index of suspicion and consider TSC even in situations of atypical presentations because early diagnosis can reduce the morbidity and mortality of the disease.

## References

[1] O'Callaghan FJ, Shiell AW, Osborne JP, Martyn CN (1998). Prevalence of tuberous sclerosis estimated by capture-recapture analysis. Lancet.

[2] Dumitrescu D, Georgescu EF, Niculescu M, Dumitrescu CI, Mogoantă SS, Georgescu I (2009). Tuberous sclerosis complex: report of two intrafamilial cases, both in mother and daughter. Rom J Morphol Embryol.

[3] Olubunmi OA (2010). Misdiagnosis of tuberous sclerosis in a Nigerian girl: a case report and review of literature. Ann Afr Med.

[4] Illahi Y, Tanveer S, Khurshid Pasha KA, Naeem A, Ali N (2010). Tuberous sclerosis: classical presentation in a male patient. NMJ.

[5] Roach ES, Gomez MR, Northrup H (1998). Tuberous sclerosis complex consensus conference: revised clinical diagnostic criteria. J Child Neurol.

[6] Randle S (2020). Tuberous sclerosis complex: genetics, clinical features, and diagnosis. UpToDate.

[7] Staley BA, Vail EA, Thiele EA (2011). Tuberous sclerosis complex: diagnostic challenges, presenting symptoms, and commonly missed signs. Pediatrics.

[8] Mostafa KG, Rasul CH, Baruri NN, Sultana R (2013). Tuberous sclerosis misdiagnosed as epilepsy. Bangladesh Med J Khulna.

